# CRT-D Therapy in Patients with Decompensated NYHA Class-Four CHF

**DOI:** 10.1155/2012/319205

**Published:** 2012-07-30

**Authors:** Faisal Zaeem, Dalia Giedriemiene, Craig Coleman, Eric Crespo, Joseph Radojevic, Steven Zweibel, Jeffrey Kluger, Christopher A. Clyne

**Affiliations:** ^1^Hartford Hospital, University of Connecticut, Hartford, CT 06102, USA; ^2^Department of Cardiology, Hartford Hospital, Hartford, CT 06102, USA; ^3^Department of Pharmacy, Hartford Hospital, Hartford, CT 06102, USA; ^4^Department of Electrophysiology, Hartford Hospital, Hartford, CT 06102, USA; ^5^CHF Department, Hartford Hospital, Hartford, CT 06102, USA

## Abstract

*Background*. ACC-HRS Guidelines for Cardiac Resynchronization Therapy ICD implantation (CRT-D) do not include patients with advanced nonambulatory NYHA class-four CHF due to an expectation of limited survival. There is little data available from these large multicenter randomized studies to support or refute this claim. *Purpose*. We evaluated the outcomes of patients with advanced nonambulatory NYHA class-four CHF who received CRT-D devices as an attempt to improve the clinical status and promote hospital discharge. *Methods*. Sixteen (of our six hundred and seventy CRT-D patients) were classified as advanced nonambulatory NYHA Class four inotrope/vasodilator/diuretic-dependent patients. These patients were analyzed retrospectively for weaning success to oral medications, hospital discharge, hemodynamic stability, and survival over eighteen months. *Results*. Thirteen of sixteen patients were discharged to home within two weeks of implantation. The survival to hospital discharge, as well as at six, twelve, and eighteen months was positive (ninety-four percent, seventy-five percent, sixty-nine percent, sixty-nine percent, resp.). The groups showed significant improvements in systolic blood pressure, renal function, left ventricular ejection fraction, and CHF class. *Conclusion*. CRT-D in advanced nonambulatory NYHA four patients proved feasible and beneficial. These findings suggest that the strategy merits further study.

## 1. Background

Chronic heart failure is a debilitating disease that continues to place an inordinate burden on the healthcare system. Management and care for the nearly five million heart failure patients in the United States alone was estimated to cost thirty-nine point two billion dollars for the year of 2010 [1, 2]. Heart failure is responsible for over one million admissions to American hospitals every year and is associated with an increased mortality [1, 2].

There have been significant contributions to the armamentarium available to physicians to help treat this disease. In addition to neurohormonal agents, cardiac resynchronization therapy (CRT) has been shown to be an effective tool in management of systolic congestive heart failure. CRT has been most effective in improving heart failure class, quality of life, left ventricular mechanics, and in reducing heart failure hospitalizations and overall mortality in patients with severe left ventricular dysfunction and a wide QRS [3, 4]. It is recognized that the beneficial impact of CRT stems from the ability of combined right ventricular and left ventricular pacing to restore both inter- and intraventricular synchrony towards normal. The result is an improvement in left ventricular geometry and mechanical function, contractility, and performance [5]. Reduction in left ventricular size and mitral regurgitation (when present) is also important for many patients [5]. Patients who benefit most are those with ejection fractions less than thirty five percent, a wide QRS (greater than one hundred twenty milliseconds), and with NYHA CHF class-three and ambulatory class-four CHF status [3, 4]. More recent studies have also demonstrated significant benefit for patients with mild NYHA class-one and class-two CHF in the presence of a left bundle branch block QRS (greater than or equal to one hundred and thirty milliseconds) and impaired left ventricular systolic function (ejection fraction less than or equal to thirty percent) [6, 7].

All of the large CRT-D trials have excluded patients on intravenous vasoactive and/or inotropic drugs [6–9]. The reasoning behind this exclusion was the presumption that these patients were too ill to benefit from CRT-D therapy given an expectedly short survival [1]. Thus, there remains a significant knowledge gap in the possible application of CRT-D therapy to patients with advanced, nonambulatory NYHA class-four CHF. We undertook this study to better assess the feasibility and outcome of CRT-D in patients with advanced nonambulatory NYHA class-four heart failure.

## 2. Methods

This was a retrospective study based on a single tertiary care center's experience. The Hartford Hospital Arrhythmia Service Database followed a total of fourteen hundred and thirty-four patients. The database included six hundred and seventy patients who had received CRT-D devices from December of two thousands through March of two thousand and nine. Sixteen patients were identified from these sixhundred and seventy as having been on intravenous inotropes, and/or vasodilators in combination with intravenous diuretics at the time of CRT-D implantation. These charts were then manually reviewed to define the patient's clinical status at the time of CRT-D implantation. Inability to wean from these intravenous drugs was confirmed and patients were further characterized (Table 1). Their clinical courses were followed in hospital using the inpatient record, and over 18 months (or until death) every three months through the Hartford Hospital ICD clinic database.

Weaning from intravenous medications was attempted on a daily basis. “Weaning success” was defined by transition from intravenous inotropes, vasodilators, and/or diuretics to oral medications, permitting patients to be discharged from the intensive care unit to an ambulatory cardiac bed. These patients were then followed for the next 18 months. Failure to maintain blood pressure, oxygen saturation, renal function (BUN, and creatinine), or recurrent heart failure symptoms requiring a return to previously effective intravenous medication doses was regarded at “failure to wean.” “Failure to wean” patients were not eligible for transfer to a usual monitored floor bed or for discharge to home. 

Patients who qualified for a CRT-D device by having a left ventricular ejection fraction of less than thirty five percent and a QRS of greater than one hundred and twenty milliseconds were offered a CRT-D device despite their nonambulatory advanced NYHA class-four status. They were aware that there were minimal data to support the use of such a device in patients with advanced CHF, but there was hope that left ventricular mechanics might improve, advancing their care to ambulatory status. Patients with systolic blood pressures of greater than ninety mmHg with an oxygen saturation of greater than ninety-five percent (with or without supplemental oxygen support) and creatinines of less than two point five milligrams percent were offered the procedure as part of our clinical practice. 

**Figure 1:**
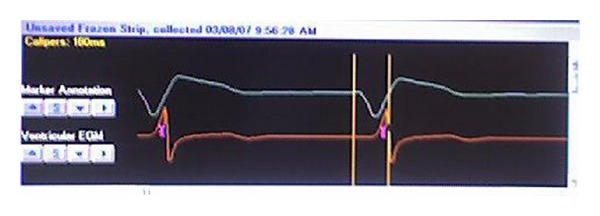
Cardiac electrogram measuring electrical separation (ES). Vertical lines measure ES from the beginning of the native QRS to the intrinsicoid deflection of the unipolar left ventricular electrogram in its final position (ES = 160 ms).

All patients were in normal rhythm and received an atrial lead. All leads were implanted transvenously. The left ventricular lead was positioned within a lateral branch of the coronary sinus. The optimal position was chosen after performing biplane coronary sinus venography and by choosing a position with the greatest temporal separation measured from the onset of the QRS to the intrinsicoid deflection of the unipolar left ventricular electrocardiogram (Figure 1). The right ventricular ICD/pacing lead was positioned in the right ventricular apex or apical septum in all patients. All patients remained in the coronary care unit until the intravenous medications could be withdrawn. Inotropes and vasodilators were progressively reduced and then discontinued as the hemodynamic status of the patient permitted. Intravenous diuretics were changed to an equivalent oral (total milligram) dose after implantation. Patients were transferred from the coronary care unit to a monitored bed as soon as continuous blood pressure recording and intubation were not required, and intravenous medications could be transitioned to oral medications. Oral medications were optimized prior to hospital discharge. Patients returned for clinical assessment and device followup clinic at two weeks, one month, and every three months thereafter. All visits were recorded in our ICD database.

## 3. Results 

The study population had a predominance of men in their late sixties with ischemic cardiomyopathies. The mean ejection fraction was fourteen point seven percent, and the mean QRS duration was one hundred and sixty-four milliseconds. All patients had a left bundle branch block QRS. A significant sixty-three percent of patients had concomitant renal disease (Table 1). 

Survival to hospital discharge was seen in ninety-four percent of the patients (fifteen of sixteen). The single predischarge death was due to significant comorbidities including acute renal failure and ventricular tachycardia. Two patients could not be weaned from intravenous inotropes and diuretics and were transferred to hospice goals of care. Thirteen of the sixteen patients (eighty-one percent) were successfully transitioned to oral medications and discharged to home in eleven point four days (plus or minus nine point four days, Table 2). Twelve patients (seventy-five percent) were alive at six months, and eleven patients (sixty-nine percent) were alive and living at home at both twelve and eighteen months after implantation. Five of the sixteen patients (thirty-one percent) received appropriate therapy for sustained ventricular tachycardia during followup. There were also significant improvements in the hemodynamic profile and patient status: systolic blood pressure increased 17.4 millimeters of mercury (*P* = 0.013), creatinine declined 0.63 milligrams per deciliter (*P* = 0.04); BUN declined 18.3 per deciliter (*P* = 0.01, and New York Heart Association Functional Class improved by 0.7 (*P* = 0.014, Table 2). 

## 4. Discussion

 Multiple CRT trials have shown improvement in left ventricular mechanics, congestive heart failure symptoms, patient quality of life, and survival in patients with advanced NYHA class-three and ambulatory class-four heart failure. Patients included in these studies also had significant left ventricular dysfunction (LVEF less than or equal to thirty-five percent) and a wide QRS [3, 4]. Similar benefits have more recently been shown in patients with less severe (NYHA class-one and two) CHF, a left ventricular ejection fraction of less than thirty percent, with a left bundle branch block pattern QRS of greater than one thirty milliseconds [6, 10]. In MADIT CRT, the single criterion of a left bundle branch block QRS separated those who gleaned benefit from CRT-D from those who did not benefit [6]. It was reasoned that left bundle branch QRS morphology is a marker in many patients for left ventricular dys-synchrony. Dys-synchrony, when present, may be rectified by resynchronization, which can improve cardiac performance and clinical status. Patients with decompensated NYHA class-four CHF have been excluded from these major studies despite having a wide QRS and a left ventricular ejection fraction of less than or equal to thirty-five percent [1, 6, 10].

A few small studies have evaluated CRT as a possible weaning/salvage therapy in this very ill population. They have demonstrated the possibility of CRT support enabling successful weaning from intravenous inotropes in hospitalized NYHA class-four CHF patients [11–14]. The largest study to date included twenty inotrope-dependent patients who were treated with CRT devices as an attempt to provide additional hemodynamic support for weaning [11]. Three of these patients had CRT-D devices. The authors report a remarkable clinical recovery after CRT with cessation of catecholamine support in all twenty patients. There was a six-month survival of eighty percent, with a fifty-five percent eighteen-month survival [11].

Our study differs from this study by Milliez and colleagues. Our sixty-nine percent eighteen month survival stands out against the fifty-five percent eighteenmonth survival reported by Milliez et al. [11] It is possible that the Milliez study patients were more ill than our patients. Three of the deaths in the Milliez study were sudden and occurred in patients without an ICD, while all of our patients received CRT-D devices. Five of our sixteen patients (thirty-one percent) received appropriate (antitachycardia pacing and/or shock) therapy for sustained ventricular tachycardia during followup. It is possible that some of the deaths seen in the Milliez study were due to cardiac arrhythmias in patients who had not received CRTD devices. 

Studies performed at other centers have demonstrated favorable outcomes for inotrope-dependent patients who were treated with cardiac resynchronization pacing therapy enabling weaning from intravenous inotropes to oral medications [11–14]. Our study agrees with the results of these other small reports and expands on the potential usefulness and effectiveness of CRT-D implantation in this population.

## 5. Conclusion

Our study reports on the survivability and effectiveness of CRT-D therapy in a patient population typically excluded from CRT-D studies. Ninety-four percent of patients were discharged from hospital, while eighty percent were discharged to home care, and sixty-nine percent of patients remained alive and home at both twelve and eighteen months after implantation. It is significant that almost one-third (thirty-one percent) of our patients received appropriate therapy for sustained ventricular tachycardia from their devices during the eighteen-month followup. These results emphasize the effectiveness of CRT-D therapy in improving hemodynamics and permitting a transition from an intravenous dependent hospital-bound status, to an ambulatory dischargeable status in some patients. Survival in out patients was sixty nine percent at eighteen months.

Despite the small size and retrospective nature of our study, we hope that this will serve as a catalyst for larger randomized controlled studies to better understand the role of CRTD therapy in patients with decompensated advanced NYHA class-four CHF.

## Figures and Tables

**Table 1:** Baseline demographics.

Characteristics	Values
Total patients	16 (100%)
Male	10 (63%)
Age (years)	68.1 ± 13.1
LVEF (%)	14.7 ± 5.9
LVEDd (cm)	6.0 ± 0.7
QRS (ms)	164.1 ± 28.2
Ischemic cardiomyopathy	11 (69%)
Impaired renal function (SCr > 1.5 mg/dL)	10 (63%)
Beta blockers	10 (63%)
Ace-inhibitors	10 (63%)
Angiotensin receptor blockers	2 (12%)
Intravenous diuretics	16 (100%)
Intravenous inotropes	13 (81%)
Dobutamine	12 (75%)
Milrinone	7 (44%)
Dopamine	1 (6%)
Nesiritide	3 (19%)

**Table 2:** Results.

Clinical variables	Pre-CRT	Post-CRT	*P* value
Systolic BP (mmHg)	92.6 ± 11.2	110.0 ± 15.8	0.0013^∗^
Serum Cr (mg/dL)	2.12 ± 0.96	1.49 ± 0.64	0.04^∗^
BUN (mg/dL)	55.9 ± 23.1	37.6 ± 12.8	0.011^∗^
Dependence on Inotropes/diuretics	16 (100%)	2 (11%)	0.001^∗^
NYHA FC	4.0 ± 0.0	3.3 ± 0.87	0.014^∗^
Hospital stay (Days)	14.3 ± 13.3	11.4 ± 9.16	0.48
